# Checklist of the family Anthomyiidae (Diptera) of Finland

**DOI:** 10.3897/zookeys.441.7527

**Published:** 2014-09-19

**Authors:** Verner Michelsen

**Affiliations:** 1 Natural History Museum of Denmark (Zoological Museum), Universitetsparken 15, DK-2100, Copenhagen Ø, Denmark

**Keywords:** Checklist, Finland, Diptera, Anthomyiidae

## Abstract

An updated checklist of the the genera and species of Anthomyiidae (Diptera) found in Finland is provided.

## Introduction

The family Anthomyiidae is a large and taxonomically difficult group of flies that has for the same reason suffered from unstable taxonomy and nomenclature. A checklist of the anthomyiid species known from pre-war Finland was compiled by their leading regional specialist of calyptrate flies Lauri Tiensuu (1906−1980) and published in Frey et al. (1941). The Anthomyiidae were then not recognized as a separate family but combined with the fanniid and true muscid flies in a comprehensive Muscidae family equivalent of the present Muscoidea less Scathophagidae. Tiensuu’s list included confirmed records of 199 anthomyiid species classified in 41 genera and subgenera. No less than 34% of the species names and 58% of the genus-group names in that list are presently replaced by other names for taxonomic and/or nomenclatorial reasons. Most changes were introduced by Hennig (1966−1976) and Michelsen (1985).

The next Finnish checklist of Anthomyiidae by Hackman (1980) was prepared under the influence by two major events. Firstly, 10% of the area of pre-war Finland, notably a major part of Finnish Karelia, had been ceded to the Soviet Union. In consequence, Hackman’s list included fewer species: 175 (too few actually, as he missed a few species recorded by Tiensuu (1936) from present Finland). Secondly, the appearance of Hennig’s (1966−1976) major revision of Palearctic Anthomyiidae had resulted in numerous name changes and redefinitions of both species and genera. Hackman’s list was updated according to Hennig’s proposals except for the splitting of newly recognized species complexes.

After the appearance of the 1941-checklist, L. Tiensuu had stopped publishing taxonomic and faunistic papers on Finnish Diptera altogether, although he stayed active all his life as a skilled collector of Finnish Diptera. Apart from W. Hennig’s study of some type specimens in the Helsinki Museum and a project on mushroom feeding *Pegomya* (Hackman 1976, 1979; Hackman and Meinander 1979) Finnish anthomyiids were given little attention until the present author in the early 1980’s began a long-term study of the species composition and distributionof Anthomyiidae in Fennoscandia and Denmark.

The web-based database *Fauna Europaea* ver. 1.0 released in 2004 included anthomyiid checklists (Michelsen 2004) for all parts of Europe. It was based extensively on literature records, but in respect to Finland and the other Scandinavian countries almost exclusively on personally verified records. The Diptera collection of the Finnish Museum of Natural History, University of Helsinki (MZH) provided most of the data on the Finnish anthomyiid fauna. After several updates, most recently ver. 2.4 from January 2011, the Finnish list of Anthomyiidae (Michelsen 2011) had grown substantially to 273 species (http://www.faunaeur.org/). The anthomyiid checklist given below has been further expanded and now contains 289 species. Comparisons with the better known anthomyiid fauna of Sweden and the presence of some species awaiting description suggest that the anthomyiid fauna of Finland may totally comprise 315−325 species.

All nomenclatorial and taxonomic adjustments (new synonyms, misidentifications, changed circumscriptions of species and genera) made since the appearance of Hackman’s (1980) checklist are indicated in the following, updated checklist. In agreement with *Fauna Europaea* the genera are listed alphabetically, as existing attempts to split the family into subfamilies and tribes are preliminary and in part poorly substantiated.

**Number of described species:**

World: Presently *c.* 2000.

Europe: 508 (Michelsen 2011)

Finland: 289 (present paper)

Faunistic knowledge level in Finland: average

## Checklist

superfamily Muscoidea Latreille, 1802

family **ANTHOMYIIDAE** Robineau-Desvoidy, 1830

***ADIA*** Robineau-Desvoidy, 1830

= ***Paregle*** auct. p. p.

*Adia cinerella* (Fallén, 1825)

***ALLIOPSIS*** Schnabl & Dziedzicki, 1911

= ***Paraprosalpia*** Villeneuve, 1922

*Alliopsis albipennis* (Ringdahl, 1928)

*Alliopsis aldrichi* (Ringdahl, 1934)

*Alliopsis atronitens* (Strobl, 1893)

*Alliopsis benanderi* (Ringdahl, 1926)

*Alliopsis billbergi* (Zetterstedt, 1838)

*Alliopsis brunneigena* (Schnabl, 1915)

= *incisa* Ringdahl, 1926

*Alliopsis conifrons* (Zetterstedt, 1845)

*Alliopsis denticauda* (Zetterstedt, 1838)

*Alliopsis dentiventris* (Ringdahl, 1918)

*Alliopsis fractiseta* (Stein, 1908)

*Alliopsis glacialis* (Zetterstedt, 1845)

*Alliopsis longipennis* (Ringdahl, 1918)

*Alliopsis maculifrons* (Zetterstedt, 1838)

*Alliopsis moerens* (Zetterstedt, 1838)

*Alliopsis sepiella* (Zetterstedt, 1845)

*Alliopsis silvestris* (Fallén, 1824)

*Alliopsis teriolensis* (Pokorny, 1893)

= *borealis* Stein, 1916

***ANTHOMYIA*** Meigen, 1803

= ***Craspedochoeta*** Macquart, 1851

= ***Craspedochaeta*** Scudder, 1884 (emend.)

= ***Chelisia*** Rondani, 1856

*Anthomyia canningsi* Griffiths, 2001

*Anthomyia confusanea* Michelsen *in* Michelsen & Báez, 1985

= *pullula* auct. p. p.

*Anthomyia imbrida* Rondani, 1866

= *pluvialis* auct. p. p.

*Anthomyia liturata* (Robineau-Desvoidy, 1830)

= *pullula* Zetterstedt, 1845

*Anthomyia mimetica* (Malloch, 1918)

= *angulata* Tiensuu, 1938

*Anthomyia monilis* (Meigen, 1826)

*Anthomyia plurinotata* Brullé, 1832

*Anthomyia pluvialis* (Linnaeus, 1758)

*Anthomyia procellaris* Rondani, 1866

= *pluvialis* auct. p. p.

***BOREOPHORBIA*** Michelsen, 1987

*Boreophorbia hirtipes* (Stein, 1907)

***BOTANOPHILA*** Lioy, 1864

= ***Pegohylemyia*** Schnabl, 1911

*Botanophila apiciseta* (Ringdahl, 1933)

*Botanophila betarum* (Lintner, 1883)

= *macra* Karl, 1940

*Botanophila biciliaris* (Pandellé, 1900)

*Botanophila bidens* (Ringdahl, 1933)

*Botanophila brunneilinea* (Zetterstedt, 1845)

*Botanophila cognata* Suwa, 2011

= *subnitida* Malloch, 1920?

*Botanophila discreta* (Meigen, 1826)

= *striolata* auct. p. p.

*Botanophila dissecta* (Meigen, 1826)

*Botanophila estonica* (Elberg, 1970)

= *varicolor* auct. p. p.

*Botanophila fugax* (Meigen, 1826)

*Botanophila gemmata* (Zetterstedt, 1860)

*Botanophila gnava* (Meigen, 1826)

*Botanophila hucketti* (Ringdahl, 1935)

*Botanophila impudica* (Rondani, 1866)

= *varicolor* auct. p. p.

*Botanophila laterella* (Collin, 1967)

*Botanophila latifrons* (Zetterstedt, 1845)

= *humeralis* Hennig, 1970

*Botanophila lobata* (Collin, 1967)

*Botanophila maculipes* (Zetterstedt, 1845)

= *pseudomaculipes* Strobl, 1893

*Botanophila miniatura* (Huckett, 1965)

*Botanophila nigra* Michelsen, 2009

*Botanophila petrophila* (Ringdahl, 1926)

*Botanophila phrenione* (Séguy, 1937)

*Botanophila profuga* (Stein, 1916)

*Botanophila relativa* (Huckett, 1965)

*Botanophila rubrifrons* (Ringdahl, 1933)

*Botanophila rubrigena* (Schnabl, 1915)

*Botanophila salicis* (Ringdahl, 1918)

*Botanophila sericea* (Malloch, 1920)

= *obscura* auct. nec Macquart, 1835

*Botanophila silvatica* (Robineau-Desvoidy, 1830)

*Botanophila sonchi* (Hardy, 1872)

*Botanophila spinosa* (Rondani, 1866)

*Botanophila striolata* (Fallén, 1824)

*Botanophila trapezina* (Zetterstedt, 1845)

= *varicolor* auct. p. p.

*Botanophila unicolor* (Ringdahl, 1932)

*Botanophila varicolor* (Meigen, 1826)

*Botanophila verticella* (Zetterstedt, 1838)

= *lineatula* Karl, 1928

***CALYTHEA*** Schnabl & Dziedzicki, 1911

*Calythea nigricans* (Robineau-Desvoidy, 1830)

***CHIASTOCHETA*** Pokorny, 1889

*Chiastocheta dentifera* Hennig, 1953

*Chiastocheta inermella* (Zetterstedt, 1838)

= *trollii* auct. p. p.

*Chiastocheta macropyga* Hennig, 1953

*Chiastocheta trollii* (Zetterstedt, 1845)

***CHIROSIA*** Rondani, 1856

= ***Craspedochaeta*** auct. p. p.

*Chirosia albitarsis* (Zetterstedt, 1845)

*Chirosia betuleti* (Ringdahl, 1935)

*Chirosia bisinuata* (Tiensuu, 1939)

*Chirosia cinerosa* (Zetterstedt, 1845)

*Chirosia crassiseta* Stein, 1908

*Chirosia flavipennis* (Fallén, 1823)

*Chirosia griseifrons* (Séguy, 1923)

*Chirosia grossicauda* Strobl, 1901

= *parvicornis* auct. nec Zetterstedt, 1845

*Chirosia histricina* (Rondani, 1866)

= *hystrix* Brischke, 1880

*Chirosia nigripes* Bezzi, 1895

= *albifrons* Tiensuu, 1938

*Chirosia similata* (Tiensuu, 1939)

***DELIA*** Robineau-Desvoidy, 1830

*Delia abruptiseta* (Ringdahl, 1935)

*Delia albula* (Fallén, 1825)

*Delia angustaeformis* (Ringdahl, 1933)

*Delia angustifrons* (Meigen, 1826)

*Delia antiqua* (Meigen, 1826)

*Delia brassicaeformis* (Ringdahl, 1926)

*Delia brunnescens* (Zetterstedt, 1845)

*Delia cardui* (Meigen, 1826)

*Delia carduiformis* (Schnabl in Schnabl & Dziedzicki, 1911)

*Delia coarctata* (Fallén, 1825)

*Delia coarctoides* Michelsen, 2007

*Delia cregyoglossa* (Huckett, 1965)

= *turcica* Hennig, 1974

*Delia criniventris* (Zetterstedt, 1860)

*Delia cuneata* Tiensuu, 1946

*Delia diluta* (Stein, 1916)

*Delia echinata* (Séguy, 1923)

*Delia fabricii* (Holmgren, 1872)

*Delia flavogrisea* (Ringdahl, 1926)

*Delia floralis* (Fallén, 1824)

*Delia florilega* (Zetterstedt, 1845)

*Delia frontella* (Zetterstedt, 1838)

*Delia hirtitibia* (Stein, 1916)

*Delia judicariae* (Pokorny, 1893)

= *flavifrons* auct. p. p.

*Delia lamelliseta* (Stein, 1900)

*Delia linearis* (Stein, 1898)

= *flabellifera* Pandellé, 1900

*Delia lineariventris* (Zetterstedt, 1845)

*Delia longicauda* (Strobl, 1898)

*Delia lophota* (Pandellé, 1900)

= *nuda* Strobl, 1901

*Delia martini* Griffiths, 1993

*Delia nigrescens* (Rondani, 1877)

*Delia nudicosta* (Ringdahl, 1949)

*Delia pallipennis* (Zetterstedt, 1838)

= *candens* Zetterstedt, 1845

*Delia penicilliventris* Ackland, 2010

= *penicillaris* auct. nec Rondani, 1866

*Delia pilifemur* (Ringdahl, 1933)

*Delia piliventris* (Pokorny, 1889)

*Delia planipalpis* (Stein, 1898)

*Delia platura* (Meigen, 1826)

*Delia pruinosa* (Zetterstedt, 1845)

= *flavifrons* Zetterstedt, 1860

*Delia radicum* (Linnaeus, 1758)

= *brassicae* Wiedemann, 1817

*Delia rimiventris* Michelsen, 2007

*Delia rondanii* (Ringdahl, 1918)

*Delia setigera* (Stein, 1920)

*Delia sileni* Michelsen, 2012

= *flavifrons* auct. p. p.

*Delia subalpina* (Ringdahl, 1926)

*Delia tarsata* (Ringdahl, 1918)

*Delia tenuiventris* (Zetterstedt, 1860)

= *angustitarsis* Malloch, 1920

*Delia tumidula* (Ringdahl, 1949)

*Delia uniseriata* (Stein, 1914)

***EGLE*** Robineau-Desvoidy, 1830

*Egle atomaria* (Zetterstedt, 1845)

*Egle brevicornis* (Zetterstedt, 1838)

*Egle ciliata* (Walker, 1849)

= *muscaria* auct. nec Fabricius, 1794

*Egle concomitans* (Pandellé, 1900)

*Egle ignobilis* Michelsen, 2009

*Egle inermis* Ackland, 1970

*Egle lyneborgi* Ackland & Griffiths in Griffiths, 2003

*Egle minuta* (Meigen, 1826)

*Egle parva* Robineau-Desvoidy, 1830

*Egle parvaeformis* Schnabl in Schnabl & Dziedzicki, 1911

*Egle pilitibia* (Ringdahl, 1918)

*Egle rhinotmeta* (Pandellé, 1900)

*Egle steini* Schnabl in Schnabl & Dziedzicki, 1911

*Egle subarctica* (Huckett, 1965)

***EMMESOMYIA*** Malloch, 1917

*Emmesomyia grisea* (Robineau-Desvoidy, 1830)

= *socia* auct. p. p.

*Emmesomyia socia* (Fallén, 1825)

***EUSTALOMYIA*** Kowarz, 1873

*Eustalomyia festiva* (Zetterstedt, 1845)

*Eustalomyia hilaris* (Fallén, 1823)

*Eustalomyia histrio* (Zetterstedt, 1838)

*Eustalomyia vittipes* (Zetterstedt, 1845)

***EUTRICHOTA*** Kowarz, 1893

= ***Eremomyia*** Stein, 1898

= ***Pegomyza*** Schnabl & Dziezicki, 1911

= ***Arctopegomyia*** Ringdahl, 1938

*Eutrichota frigida* (Zetterstedt, 1845)

*Eutrichota labradorensis* (Malloch, 1920)

*Eutrichota longimana* (Pokorny, 1887)

*Eutrichota pilimana* (Ringdahl, 1918)

*Eutrichota praepotens* (Wiedemann, 1817)

*Eutrichota schineri* (Schnabl, 1910)

= *socculata* auct. nec Zetterstedt, 1845

*Eutrichota socculata* (Zetterstedt, 1845)

= *consanguinea* Tiensuu, 1938

*Eutrichota tunicata* (Zetterstedt, 1846)

***FUCELLIA*** Robineau-Desvoidy, 1842

*Fucellia fucorum* (Fallén, 1819)

*Fucellia griseola* (Fallén, 1819)

*Fucellia tergina* (Zetterstedt, 1845)

***HETEROSTYLODES*** Hennig, 1967

*Heterostylodes macrura* (Schnabl in Schnabl & Dziedzicki, 1911)

*Heterostylodes nominabilis* (Collin, 1947)

= *pratensis* auct. p. p.

*Heterostylodes obscura* (Macquart, 1835)

= *pratensis* auct. p. p.

*Heterostylodes pilifera* (Zetterstedt, 1845)

= *pratensis* auct. p. p.

*Heterostylodes pratensis* (Meigen, 1826)

***HYDROPHORIA*** Robineau-Desvoidy, 1830

*Hydrophoria lancifer* (Harris, 1780)

= *conica* Wiedemann, 1817

*Hydrophoria linogrisea* (Meigen, 1826)

*Hydrophoria silvicola* (Robineau-Desvoidy, 1830)

= *annulata* auct. nec Pandellé, 1899

***HYLEMYA*** Robineau-Desvoidy, 1830

*Hylemya nigrimana* (Meigen, 1826)

*Hylemya urbica* Wulp, 1896

= *latifrons* Schnabl in Schnabl & Dziedzicki, 1911

*Hylemya vagans* (Panzer, 1798)

= *strenua* Robineau-Desvoidy, 1830

*Hylemya variata* (Fallén, 1823)

***HYLEMYZA*** Schnabl & Dziedzicki, 1911

= ***Hylemya*** auct. p. p.

*Hylemyza partita* (Meigen, 1826)

***LASIOMMA*** Stein, 1916

= ***Crinurina*** Karl, 1928

= ***Acrostilpina*** Ringdahl, 1929

*Lasiomma anthomyinum* (Rondani, 1866)

*Lasiomma atricauda* (Zetterstedt, 1845)

*Lasiomma collini* (Ringdahl, 1929)

*Lasiomma craspedodontum* (Hsue, 1980)

*Lasiomma cuneicorne* (Zetterstedt, 1838)

*Lasiomma iwasai* Suwa, 1978

*Lasiomma japonicum* Suwa, 1971

*Lasiomma latipenne* (Zetterstedt, 1838)

*Lasiomma monticola* Suh & Kwon, 1985

*Lasiomma morionellum* (Zetterstedt, 1838)

*Lasiomma picipes* (Meigen, 1826)

= *octoguttata* Zetterstedt, 1845

*Lasiomma quinquelineatum* (Ringdahl, 1926)

*Lasiomma seminitidum* (Zetterstedt, 1845)

*Lasiomma strigilatum* (Zetterstedt, 1838)

= *nitidicauda* Zetterstedt, 1855

***LEUCOPHORA*** Robineau-Desvoidy, 1830

*Leucophora cinerea* Robineau-Desvoidy, 1830

*Leucophora grisella* Hennig, 1967

*Leucophora obtusa* (Zetterstedt, 1838)

*Leucophora sericea* Robineau-Desvoidy, 1830

*Leucophora tavastica* (Tiensuu, 1939)

*Leucophora unilineata* (Zetterstedt, 1838)

*Leucophora unistriata* (Zetterstedt, 1838)

***MYCOPHAGA*** Rondani, 1856

*Mycophaga testacea* (Gimmerthal, 1834)

***MYOPINA*** Robineau-Desvoidy, 1830

*Myopina myopina* (Fallén, 1824)

= *reflexa* Robineau-Desvoidy, 1830

*Myopina scoparia* (Zetterstedt, 1845)

***PARADELIA*** Ringdahl, 1933

= ***Pseudonupedia*** Ringdahl, 1959 (*nomen nudum*)

= ***Pseudonupedia*** Huckett, 1971

*Paradelia brunneonigra* (Schnabl in Schnabl & Dziedzicki, 1911)

*Paradelia intersecta* (Meigen, 1826)

*Paradelia lunatifrons* (Zetterstedt, 1846)

*Paradelia lundbeckii* (Ringdahl, 1918)

***PAREGLE*** Schnabl, 1911

= ***Chionomyia*** Ringdahl, 1933

*Paregle atrisquama* (Ringdahl, 1948)

*Paregle audacula* (Harris, 1780)

= *radicum* auct. nec Linnaeus, 1758

*Paregle vetula* (Zetterstedt, 1838)

***PEGOMYA*** Robineau-Desvoidy, 1830

*Pegomya atricauda* Ringdahl, 1944

*Pegomya avida* Hennig, 1973

*Pegomya betae* (Curtis, 1847)

= *hyoscyami* auct. p. p.

*Pegomya bicolor* (Wiedemann, 1817)

*Pegomya caesia* Stein, 1906

*Pegomya calyptrata* (Zetterstedt, 1846)

*Pegomya circumpolaris* Ackland & Griffiths in Griffiths, 1983

*Pegomya conformis* (Fallén, 1825)

*Pegomya cunicularia* (Rondani, 1866)

= *hyoscyami* auct. p. p.

*Pegomya curviphallis* Michelsen, 2006

*Pegomya deprimata* (Zetterstedt, 1845)

*Pegomya exilis* (Meigen, 1826)

= *hyoscyami* auct. p. p.

*Pegomya flavifrons* (Walker, 1849)

*Pegomya flavoscutellata* (Zetterstedt, 1838)

*Pegomya fulgens* (Meigen, 1826)

*Pegomya furva* Ringdahl, 1938

*Pegomya fuscinata* Tiensuu, 1939

*Pegomya geniculata* (Bouché, 1834)

*Pegomya grahami* Michelsen & Ackland, 2009

*Pegomya haemorrhoum* (Zetterstedt, 1838)

= *haemorrhoa* (misspelling)

*Pegomya holmgreni* (Boheman, 1859)

*Pegomya holosteae* (Hering, 1924)

*Pegomya hyoscyami* (Panzer, 1809)

*Pegomya incisiva* Stein, 1906

*Pegomya interruptella* (Zetterstedt, 1855)

*Pegomya lurida* (Zetterstedt, 1846)

*Pegomya maculata* Stein, 1906

= *atricauda* auct. p. p.

*Pegomya meridiana* (Villeneuve, 1923)

*Pegomya notabilis* (Zetterstedt, 1846)

= *zonata* auct. nec Zetterstedt, 1838

*Pegomya pallidoscutellata* (Zetterstedt, 1852)

*Pegomya pulchripes* (Loew, 1857)

= *flavipes* Fallén, 1825

*Pegomya rubivora* (Coquillett in Slingerland, 1897)

*Pegomya ruficeps* (Zetterstedt, 1838)

*Pegomya rufina* (Fallén, 1825)

*Pegomya scapularis* (Zetterstedt, 1846)

= *pilosa* Stein, 1900

*Pegomya seitenstettensis* (Strobl, 1880)

*Pegomya setaria* (Meigen, 1826)

*Pegomya solennis* (Meigen, 1826)

= *nigritarsis* Zetterstedt, 1838

*Pegomya steini* Hendel, 1925

*Pegomya tabida* (Meigen, 1826)

*Pegomya tenera* (Zetterstedt, 1838)

*Pegomya testacea* (De Geer, 1776)

= *silacea* Meigen, 1830

*Pegomya transgressa* (Zetterstedt, 1846)

*Pegomya vanduzeei* Malloch, 1919

= *versicolor* auct. nec Meigen, 1826

*Pegomya vittigera* (Zetterstedt, 1838)

*Pegomya winthemi* (Meigen, 1826)

*Pegomya zonata* (Zetterstedt, 1838)

= *tenera* auct. nec Zetterstedt, 1838

***PEGOPLATA*** Schnabl & Dziedzicki, 1911

= ***Nupedia*** Karl, 1930

*Pegoplata aestiva* (Meigen, 1826)

*Pegoplata annulata* (Pandellé, 1899)

= *virginea* auct. nec Meigen, 1826

*Pegoplata infirma* (Meigen, 1826)

*Pegoplata nigroscutellata* (Stein, 1920)

*Pegoplata palposa* (Stein, 1897)

*Pegoplata patellans* (Pandellé, 1900)

*Pegoplata tundrica* (Schnabl, 1915)

***PHORBIA*** Robineau-Desvoidy, 1830

*Phorbia atrogrisea* Tiensuu, 1936

*Phorbia curvicauda* (Zetterstedt, 1845)

*Phorbia fascicularis* Tiensuu, 1936

*Phorbia fumigata* (Meigen, 1826)

= *securis* Tiensuu, 1936

*Phorbia genitalis* (Schnabl in Schnabl & Dziedzicki, 1911)

*Phorbia longipilis* (Pandellé, 1900)

*Phorbia melania* Ackland & Michelsen, 1987

*Phorbia moliniaris* (Karl, 1917)

*Phorbia penicillaris* (Stein, 1916)

*Phorbia singularis* Tiensuu, 1938

***Ringdahlia*** Michelsen, 2014

*Ringdahlia curtigena* (Ringdahl, 1935)

***STROBILOMYIA*** Michelsen, 1988

= ***Lasiomma*** auct. p. p.

*Strobilomyia anthracina* (Czerny, 1906)

*Strobilomyia infrequens* (Ackland, 1965)

*Strobilomyia laricicola* (Karl, 1928)

*Strobilomyia sibirica* Michelsen, 1988

*Strobilomyia svenssoni* Michelsen, 1988

***SUBHYLEMYIA*** Ringdahl, 1933

*Subhylemyia longula* (Fallén, 1824)

***ZAPHNE*** Robineau-Desvoidy, 1830

= ***Acroptena*** Pokorny, 1893

= ***Hydrophoria*** auct. p. p.

*Zaphne ambigua* (Fallén, 1823)

*Zaphne barbiventris* (Zetterstedt, 1845)

*Zaphne brunneifrons* (Zetterstedt, 1838)

*Zaphne caudata* (Zetterstedt, 1855)

*Zaphne divisa* (Meigen, 1826)

*Zaphne fasciculata* (Schnabl, 1915)

*Zaphne frontata* (Zetterstedt, 1838)

*Zaphne ignobilis* (Zetterstedt, 1845)

*Zaphne inuncta* (Zetterstedt, 1838)

= *hyalipennis* Zetterstedt, 1855

*Zaphne lineatocollis* (Zetterstedt, 1838)

= *laticornis* Ringdahl, 1916

*Zaphne nuda* (Schnabl in Schnabl & Dziedzicki, 1911)

*Zaphne proxima* (Malloch, 1920)

*Zaphne verticina* (Zetterstedt, 1838)

*Zaphne wierzejskii* (Mik, 1867)

*Zaphne zetterstedtii* (Ringdahl, 1918)

## Excluded species

*Botanophila rotundivalva* (Ringdahl, 1937). Misdentification of closely related, still undescribed species first recorded from Ks by Frey (1956).

*Delia angustiventris* (Zetterstedt, 1845). Misdentification of *Delia nudicosta* (Ringdahl) by Tiensuu (1936).

*Delia floricola* Robineau-Desvoidy, 1830. An erroneous record introduced by Hennig (1974).

*Delia penicillosa* Hennig, 1974. An erroneous record introduced by Michelsen (2004).

*Lasiomma multisetosum* (Ringdahl, 1926). According to Tiensuu (1936) seen from ”finnischen Lappland” by O. Ringdahl, but no specimens have been found in the Museum of Zoology, Lund University.

*Pegomya glabroides* Michelsen, 2008. Listed from Finland (Kuusamo: Kuolajärvi) by Michelsen (2008), but Kuolajärvi (now Kuoloyarvi) is presently situated in Russian Murmansk Oblast close to the Finnish border.

*Pegomya laticornis* (Fallén, 1825) (= *genupuncta* Stein, 1906). Identification inferred by Hendel (1922) from finds of leaf mines on *Arctium* by Linnaniemi (1913). Supposed *Pegomya*-leaf mines on *Arctium* have been reported from Finland several times since then, but no flies have been recovered so far.

*Pegomya nigrisquama* (Stein, 1888). Identification inferred by Hering (1925) from finds of leaf-mines on *Solidago virgaurea* by Linnaniemi (1913).

*Pegomya sociella* Stein, 1906. Only recorded from Le: Kilpisjärvi (Tiensuu 1933). Probably a misidentification.

*Phorbia bartaki* Ackland & Michelsen, 1987. Records by Kahanpää (2013) refer to *Phorbia melania* Ackland & Michelsen.
